# Decreased succinate dehydrogenase B in human hepatocellular carcinoma accelerates tumor malignancy by inducing the Warburg effect

**DOI:** 10.1038/s41598-018-21361-6

**Published:** 2018-02-15

**Authors:** Po-Lin Tseng, Wei-Hsuan Wu, Tsung-Hui Hu, Chih-Wei Chen, Hung-Chi Cheng, Chien-Feng Li, Wen-Hui Tsai, Hui-Ju Tsai, Meng-Che Hsieh, Jiin-Haur Chuang, Wen-Tsan Chang

**Affiliations:** 1 Graduate Institute of Clinical Medical Sciences, College of Medicine, Chang Gung University, Taoyuan, 302 Taiwan; 2 Department of Biochemistry and Molecular Biology, College of Medicine, National Cheng Kung University, Tainan, 701 Taiwan; 3 Division of Hepato-Gastroenterology, Department of Internal Medicine, Chang Gung Memorial Hospital–Kaohsiung Medical Center, Kaohsiung, 833 Taiwan; 4 Institute of Clinical Medicine, College of Medicine, National Cheng Kung University, Tainan, 701 Taiwan; 5 Department of Pathology, Chi Mei Foundation Medical Center, Tainan, 710 Taiwan; 6 Department of Pediatrics, Chi Mei Foundation Medical Center, Tainan, 710 Taiwan; 7 Department of Pediatric Surgery, Kaohsiung Chang Gung Memorial Hospital–Kaohsiung Medical Center, Kaohsiung, 833 Taiwan; 8 Department of Surgery, Chi Mei Foundation Medical Center, Tainan, 710 Taiwan; 9 Department of Occupational Safety and Health/Institute of Industrial Safety and Disaster Prevention, College of Sustainable Environment, Chia Nan University of Pharmacy and Science, Tainan, 717 Taiwan

## Abstract

Changes in TCA cycle enzymes or respiratory activity are possible mechanisms of aerobic glycolysis that contributes to tumor progression. To clarify whether the decrease of succinate dehydrogenase B (SDHB) alters energy metabolism, induces the Warburg effect and results in tumor malignancy, SDHB expression was examined and modulated in hepatocellular carcinoma (HCC) tissues and cells, respectively. SDHB level was often decreased in malignant HCC cells and tissues. Furthermore, the reduced SDHB expression was associated with advanced tumor stage and poor survival rate. Moreover, silencing of SDHB altered energy metabolism switched from aerobic respiration to glycolysis, resulted in the Warburg effect, and enhanced cell proliferation and motility. In contrast, the SDHB overexpression deregulated bioenergetic metabolism and decreased cell growth and migration. In mouse xenograft models, subcutaneous implantation and tail vein injection with SDHB knockdown cells resulted in a larger tumor volume and accelerated cancer metastasis, respectively. A mutation or decrease in SDHB induced the switch from aerobic respiration to glycolysis. This metabolic alteration was associated with tumor cell dedifferentiation, proliferation, motility and overall patient survival in HCC.

## Introduction

Hepatocellular carcinoma (HCC) is the third most common cause of cancer-related mortality worldwide and the second most widespread type of tumor in Taiwan^1,2^. The poor long-term prognosis is caused by the rapid proliferation and metastasis of HCC cells. This malignant progression is resulted from deregulated genetic expression, such as inactivation of tumor suppressor genes (TSGs) or activation of oncogenes^3,4^. Previous study indicated that one of the putative TSGs, assumed to be located on chromosome arm 1p (Ch. 1p), might be involved in early step hepatocarcinogenesis^5^. The metabolic enzyme succinate dehydrogenase subunit B (SDHB), has been mapped to Ch. 1p36, which is a locus associated with many TSGs in a number of cancers, including HCC^6,7^. Changes in the bioenergetic metabolism have also been considered an important characteristic of HCC^8^. Thus, examining the correlation between bioenergetic changes and tumor progression is important to understand hepatic carcinogenesis and to further identify potential therapeutic targets.

SDH, an important mitochondrial enzyme encoded in the nucleus, catalyzes succinate oxidation in the tricarboxylic acid (TCA) cycle and couples electrons to ubiquinone in the respiratory chain^9^. Changes in TCA cycle enzymes or respiratory activities are possible mechanisms of aerobic glycolysis that contributes to tumorigenesis^10–12^. Recent studies revealed that inherited changes in mitochondrial SDH and fumarate hydratase (FH) induce hereditary tumors^7,13^. These loss-of-function mutations lead to an accumulation of succinate and fumarate, which activate hypoxia-inducible factor (HIF) and its downstream glycolytic pathway^14^. SDH is a heterotetrameric complex composed of four subunits, including SDHA, -B, -C and -D. Germline mutations of SDHB, -C and -D lead to pheochromocytoma or paraganglioma^15^.

SDHB, a hydrophilic subunit containing three iron-sulfur clusters, forms the key interface with the anchor proteins SDHC and -D^6,9^. SDHB may play a pivotal role in tumorigenesis through induction of HIF activity^14,16^. Mutations in SDHB occur at high incidences in adrenal and extra-adrenal pheochromocytoma and are associated with high frequencies of malignant and metastatic tumors, such as malignant pheochromocytoma and in some cases, renal cell carcinoma^17–19^. However, the biological function of the SDHB protein in tumorigenesis or malignant transformation in other solid tumors and, in particular, the loss or decrease in its expression levels has not been fully explained.

Therefore, we hypothesized that the SDHB gene might function as a TSG in the development and progression of HCC. In addition, silenced SDHB expression caused a major impairment in cell proliferation, which was demonstrated previously only in an *in vitro* model of a HCC cell line^20^. However, no detailed analysis of the clinical significance of SDHB expression levels in human HCC samples has been reported. In this study, the clinical significance of SDHB expression in HCC tumors was investigated. To elucidate whether this gene was involved in the development or progression of HCC, we created and analyzed several stable SDHB-silenced cells using RNA interference (RNAi) and established and characterized persistent and high SDHB expression in cells using an ectopic overexpression vector.

## Results

### SDHB expression is often decreased in malignant HCC cell lines and tumor tissues

**Figure 1:**
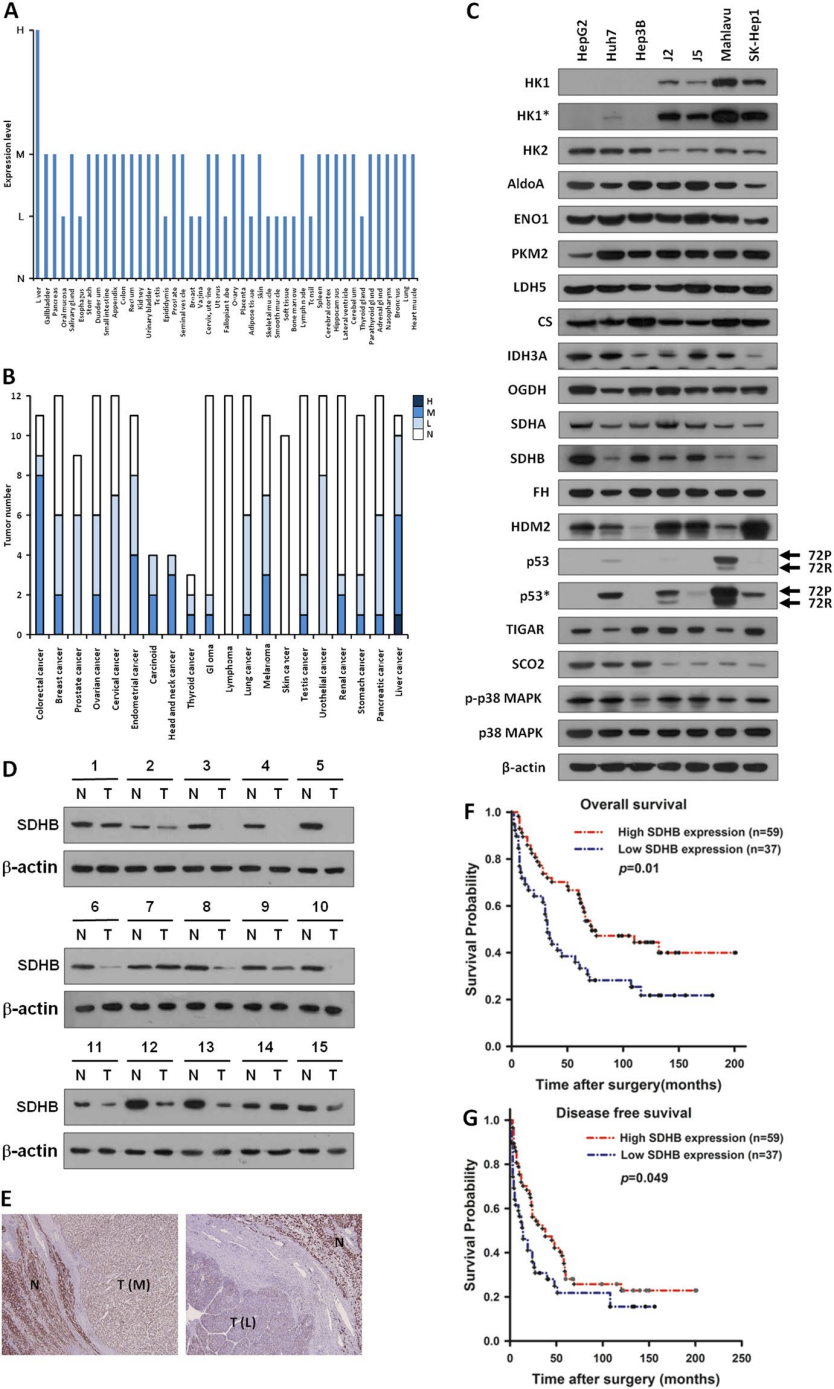
SDHB expression is often decreased in malignant HCC cell lines and tumor tissues. (**A**,**B**) Analysis of SDHB expression in human normal tissues and organs, as well as in human cancers. SDHB was searched within the Human Protein Atlas (http://www.proteinatlas.org/) website. (**C**) Western blot analysis of proteins involved in glycolysis and the TCA cycle in seven HCC cell lines. Total proteins prepared from cells as indicated were blotted with the indicated antibodies. (**D**) Western blotting of SDHB expression in 15 pairs of fresh HCC tumor and noncancerous tissues. Total proteins purified from tissues were hybridized with antibodies against SDHB and β-actin. (**E**) Immunohistochemical staining of SDHB in HCC specimens. Two surgically resected specimens were immunohistochemically stained with an antibody against SDHB. N and T indicate the normal and tumor tissues in the HCC specimens, respectively. L and M represent low and moderate expression levels of SDHB in HCC tumors, respectively. (**F**,**G**) Kaplan-Meier plot of overall and disease-free survivals of HCC patients grouped according to SDHB expression levels. Tumors with decreased SDHB expression were associated with lower overall tumor-free survival in HCC patients. The level of β-actin serves as a control for protein loading.

To understand the functional role of SDHB in biological processes, analysis of its expression pattern in all tissues and organs is needed. The SDHB was searched within the Human Protein Atlas (http://www.proteinatlas.org/) website. The results revealed that most tissues and organs exhibit low to moderate levels of the SDHB protein, with the highest expression in the liver (Fig. 1A). In addition, the preliminary analysis showed that most of the HCC tumor specimens exhibited low to moderate levels of SDHB expression, suggesting that SDHB expression is altered during hepatic carcinogenesis or tumor progression (Fig. 1B).

To establish a direct correlation between SDHB expression levels and hepatic tumorigenesis or malignancy, the SDHB protein was examined by Western blotting and immunohistochemical staining in HCC cell lines and cancer specimens. Western blot analysis was used to determine SDHB protein levels in seven HCC cell lines, including HepG2, Huh7, Hep3B, J2, J5, Mahlavu and SK-Hep1. Low SDHB expression was detected in the poorly differentiated cell lines of Mahlavu and SK-Hep1, whereas high expression was observed in the well-differentiated cell lines Hep3B and HepG2, indicating that down-regulation of SDHB expression was associated with tumor cell differentiation (Fig. 1C). Western blot analysis was also used to measure SDHB protein levels in primary HCC tumor tissues and the surrounding non-cancerous tissues. Decreased expression of SDHB was observed in 10/15 (67%) HCC specimens compared with the non-tumorous counterparts, indicating frequent down-regulated expression in HCC (Fig. 1D).

### Decreased SDHB expression is associated with advanced tumor stage and poor survival

Surgically resected specimens were collected from 96 patients, comprising 77 males and 19 females. The mean age of the patients was 55.4 ± 12.2 years. Tumor sizes ranged from 1–20 cm, with a mean size of 7.19 ± 4.15 cm. Cancer cell differentiation was classified as well (24 cases), moderate (49 cases) or poor (23 cases). In addition, for the tumor pathological stages, 14 cases were stage I, 30 stage II, 31 stage III and 21 stage IV. Furthermore, liver cirrhosis was present in 58 (60.4%) cases and non-cirrhotic livers in 38 (39.6%) cases (Table 1). Immunohistochemical staining was used to determine SDHB expression in the tumors, and weak or moderately positive SDHB staining (<50%) was observed (Fig. 1E). In 96 tumor specimens, 39 (40.6%) cases had low SDHB and 57 (59.4%) high SDHB expression in cancerous tissues. Low SDHB expression in cancer tissues was associated with tumor dedifferentiation (p < 0.01) and borderline associated with advanced pathologic stage (p = 0.06) (Table 2). Patient exhibiting low SDHB expression had poor disease-free and overall survival compared with those with high SDHB expression in tumor tissues by Kaplan-Meier analyses (Fig. 1F,G). Furthermore, the univariate analyses showed that SDHB low expression, alpha-fetoprotein (AFP), tumor capsule, tumor size, tumor grades and pathological stages were associated with both disease-free and overall survival. Multivariate analyses revealed that the correlations of AFP and tumor stages with disease-free and overall survival (Table 3).

**Table 1:** Baseline clinicopathological characteristics of HCC patients.

Variables		No (%)
Gender	Male	77 (80.2)
	Female	19 (19.8)
Viral etiology	HBsAg (+)	66 (68.8)
	Anti-HCV (+)	25 (26.0)
	HBsAg (−), Anti-HCV (−)	5 (5.2)
Cirrhosis	With	57 (59.4)
	Without	39 (40.6)
Differentiation	Well	24 (25.0)
	Moderate	49 (51.0)
	Poor	23 (24.0)
Tumor stage	I+II	44 (45.8)
	III+IV	52 (54.2)
Tumor number	Solitary	72 (75.0)
	Multiple	24 (25.0)
Capsule Intact	With	57 (59.4)
	Without	39 (40.6)
Tumor size (cm)	Mean (SD)	6.50 ± 3.78
Age (yrs)	Mean (SD)	55.4 ± 12.2
AFP(ng/ml)	Mean (SD)	2883 ± 9429
MVD	Mean (SD)	109.5 ± 41.3

HBsAg: hepatitis B surface antigen, HCV: hepatitis C virus, MVD: microvessel density, AFP: alpha-fetoprotein.

**Table 2:** Correlation between SDHB expression in tumor tissues and clinicopathologic parameters of HCCs.

Variant		SDHB expression in tumor tissues		*P* value
		High (n = 57) (%)	Low (n = 39) (%)	
Gender	Male/Female	46 (60)/11 (58)	31 (40)/8 (42)	NS
HBsAg	Positive/Negative	37 (56)/17 (63)	29 (44)/10 (37)	0.645
Anti-HCV	Positive/Negative	15 (60)/39 (57)	10 (40)/29 (43)	NS
Cirrhosis	With/Without	35 (60)/22 (56)	23 (40)/16 (44)	0.835
Differentiation	Well/ mod/ poor	19 (79)/30 (61)/8 (34.7)	5 (21)/19 (39)/15 (55.3)	0.008
Tumor stage	I, II / III, IV	31 (63)/26 (50)	13 (37)/26 (50)	0.06
Tumor No.	Solitary/Multiple	43 (59)/14 (58)	29 (41)/10(42)	NS
Capsule status	With/Without	32 (56)/25 (64)	25 (44)/14 (36)	0.527
Tumor size (cm)	(mean ± SD)	6.10 ± 3.65	7.09 ± 3.94	NS
MVD	(mean ± SD)	226.8 ± 79.5	207.5 ± 87.3	NS
Age (yrs)	(mean ± SD)	55.3 ± 12.3	55.5 ± 12.4	NS
AFP (ng/ml)	(mean ± SD)	1934 ± 9516	4307 ± 9238	NS

HBsAg: hepatitis B surface antigen, HCV: hepatitis C virus, MVD: microvessel density, AFP: alpha-fetoprotein, SD: standard deviation, NS: not significant.

**Table 3:** Univariate and multivariate Cox Regression analyses of overall and disease-free survivals of HCC patients.

Factors	Overall survival				Disease-free survival			
	Univariate		Multivariate		Univariate		Multivariate	
	HR(95%CI)	*p*	HR(95%CI)	*p*	HR(95%CI)	*p*	HR(95%CI)	*p*
**Biomarker**								
SDHB low expression	1.91 (1.15–3.16)	0.012	—	—	1.57 (1.01–2.45)	0.049	—	—
**Clinical parameters**								
Age	0.94 (0.56–1.58)	0.811	—	—	0.76 (0.47–.123)	0.264	—	—
Gender	1.52 (0.77–3.00)	0.224	—	—	1.22 (0.69–2.15)	0.487	—	—
AFP	2.12 (1.27–3.55)	0.004	1.86 (1.06–3.25)	0.03	2.65 (1.65–4.26)	0.000	3 (1.75–5.16)	0.000
HBV	1.79 (0.97–3.33)	0.064	—	—	1.23 (0.74–2.05)	0.418	—	—
HCV	0.6 (0.33–1.12)	0.109	—	—	0.72 (0.42–1.22)	0.222	—	—
Cirrhosis	1.4 (0.83–2.38)	0.213	—	—	1.31 (0.82–2.10)	0.266	—	—
**Pathology parameters**								
Tumor capsule	2.43 (0.25–0.75)	0.003	—	—	0.53 (0.33–0.86)	0.009	—	—
Tumor size	2.23 (1.32–3.77)	0.003	—	—	1.74 (1.10–2.75)	0.018	—	—
Tumor number	1.11 (0.62–1.99)	0.719	—	—	1.51 (0.95–2.59)	0.079	—	—
Pathology stages	3.35 (1.93–5.82)	0.000	2.31 (1.15–4.64)	0.018	2.48 (1.54–3.99)	0.000	2.129 (1.15–3.95)	0.017
Grades	1.8 (1.04–3.14)	0.037	—	—	1.8 (1.04–3.14)	0.045	—	—

AFP: alpha fetoprotein, HBV:hepatitis B virus; HCV: hepatitis C virus.

Age ≥60 or <60 years; Gender, male or female; serum AFP ≥400 or <400; HBV+, with or without; HCV+, with or without; Cirrhosis, with or without; Tumor capsulation, with or without; Tumor size, ≥5 or <5 cm; Tumor number, solitary or ≥2; Grades, I+II or III+IV.

### Silenced SDHB expression promotes tumor cell proliferation and migration

**Figure 2:**
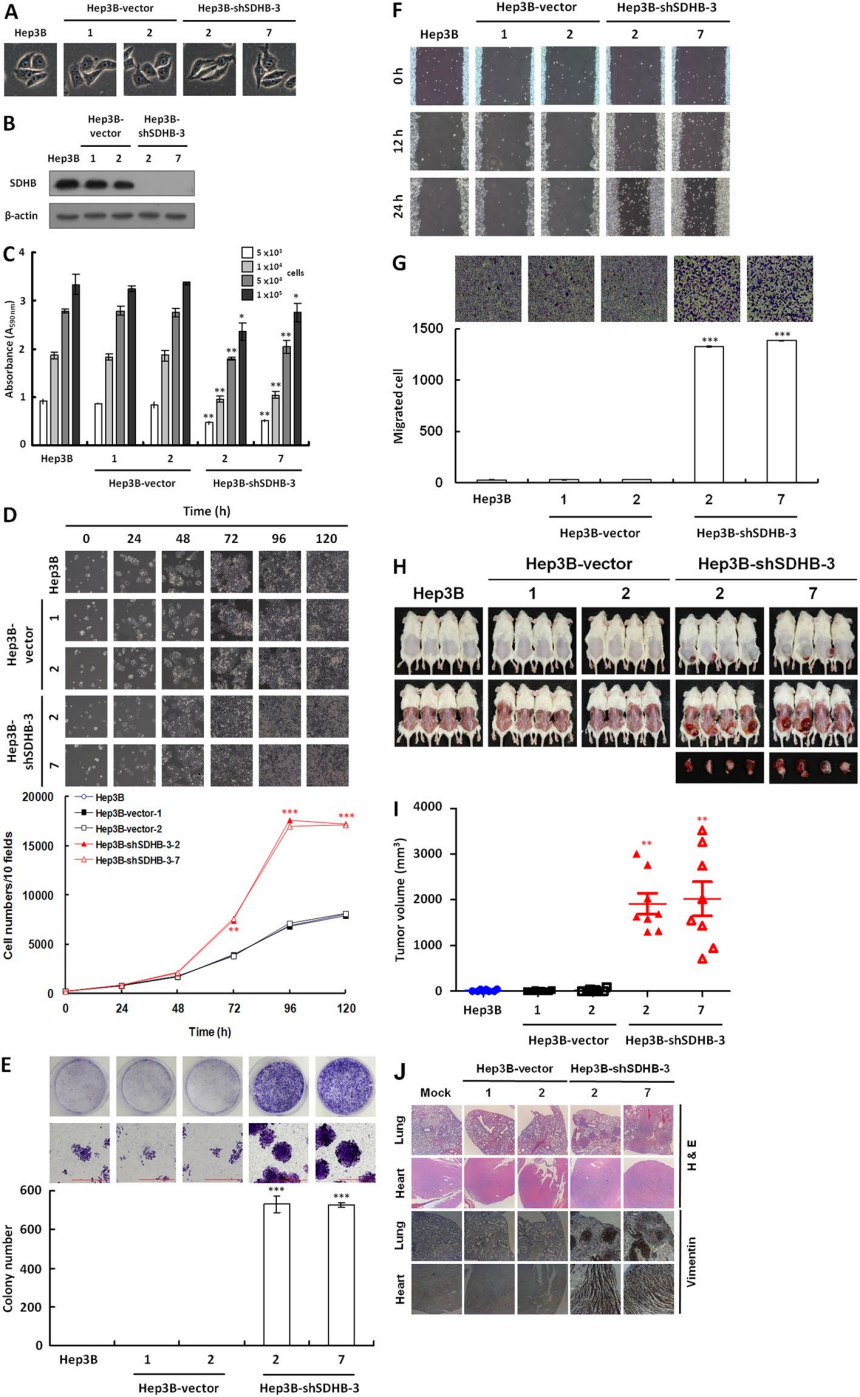
Knockdown of SDHB in HCC Hep3B cells greatly increases tumor cell proliferation and migration. (**A**) Cell morphology of SDHB-knockdown cells. Cells were grown for 48 h and imaged microscopically. (**B**) Western blot analysis of SDHB protein in SDHB-knockdown cells. Total proteins isolated from cells as indicated were blotted with antibodies against SDHB and β-actin. (**C**) SDH activity assay in SDHB-knockdown cells. Cells of equal-numbers, as indicated, were subjected to a MTT assay according to standard protocols. (**D**) SDHB-knockdown cell counts. Cells as indicated were cultured in 6-well plates, and 10 random fields were imaged microscopically. Cells in the 10 fields were scored. (**E**) Colony formation assay of SDHB-knockdown cells. Cells as indicated were seeded into 6-well plates for 7 days. The colonies obtained were stained and scored. (**F**) Wound healing migration assay of SDHB-knockdown cells. Cells as indicated were grown until confluent before a scratch wound healing migration assay was performed. (**G**) Boyden chamber migration assay of SDHB-knockdown cells. Cells as indicated were plated in a Boyden chamber and incubated for 8 h. The migrated cells were stained and counted. (**H**) *In vivo* tumor growth assay of SDHB-knockdown cells. Cells as indicated were subcutaneously inoculated into the back of NOD/SCID mice for 14 days. The mice were sacrificed and the tumors removed and examined. (**I**) Tumor volumes were measured after removal. SDHB-knockdown cells exhibited a larger tumor volume compared with mock and vector-transfected cells. (**J**) Histological and immunohistochemical staining of the lung and heart in the *in vivo* tumor metastasis assay. Cells as indicated were intravenously injected into the tail vein of NOD/SCID mice for 20 days. Mice were sacrificed and examined for tumor metastasis. Experiments were carried out using H&E staining and an antibody specific for vimentin. The β-actin level serves as a control for protein loading. The *^,^ ** and *** represented *P*-value < 0.05, <0.01 and <0.005, respectively.

To determine whether SDHB plays a pivotal role during tumor progression and malignancy, we evaluated the effect of SDHB inhibition using RNAi on HCC Hep3B cells (Fig. 2A). Compared with the mock- and vector-transfected cells, a shSDHB-3-specific shRNA was used to knockdown SDHB and resulted in >80% inhibition of SDHB protein levels (Fig. 2B). The silencing effect was confirmed using a MTT cell growth assay, in which the SDHB-knockdown cells exhibited low SDH activity compared with the mock- and vector-transfected cells (Fig. 2C). Cell count results showed that SDHB-knockdown significantly enhanced cell proliferation (Fig. 2D). To examine the effect of SDHB knockdown on cancer cell colonization, a colony formation assay was performed. Anchorage-dependent clonogenic growth was greatly enhanced, resulting in increased colony size and numbers of SDHB-knockdown cells (Fig. 2E). Furthermore, in a wound healing migration assay, cell confluence was reached sooner in the SDHB-knockdown cells than in the mock- and vector-transfected cells (Fig. 2F). A Boyden chamber invasion assay revealed that the number of invading SDHB-knockdown cells was significantly higher than those of mock- and vector-transfected cells (Fig. 2G). To confirm the effects of SDHB knockdown on HCC Hep3B cells, we repeated the experiments using HCC HepG2 and Huh7 cells. The results reveled that silenced SDHB expression increased cell proliferation and migration in both HepG2 and Huh7 cells (Figs S1 and S2). To evaluate the knockdown effect of SDHB on tumor growth and metastasis *in vivo*, the SDHB knockdown Hep3B cells implanted subcutaneously or injected via tail vein into non-obese diabetic/severe combined immunodeficient (NOD/SCID) mice were examined. The SDHB-knockdown cells showed increased tumor formation and volume, as well as accelerated cancer cell metastasis compared with the mock- and vector-transfected cells (Fig. 2H–J). To characterize the behaviors and features of SDHB-knockdown cells, we specifically chosen SDHB knockdown Hep3B cells for further analyses.

### Knockdown of SDHB expression induces bioenergetic changes

**Figure 3:**
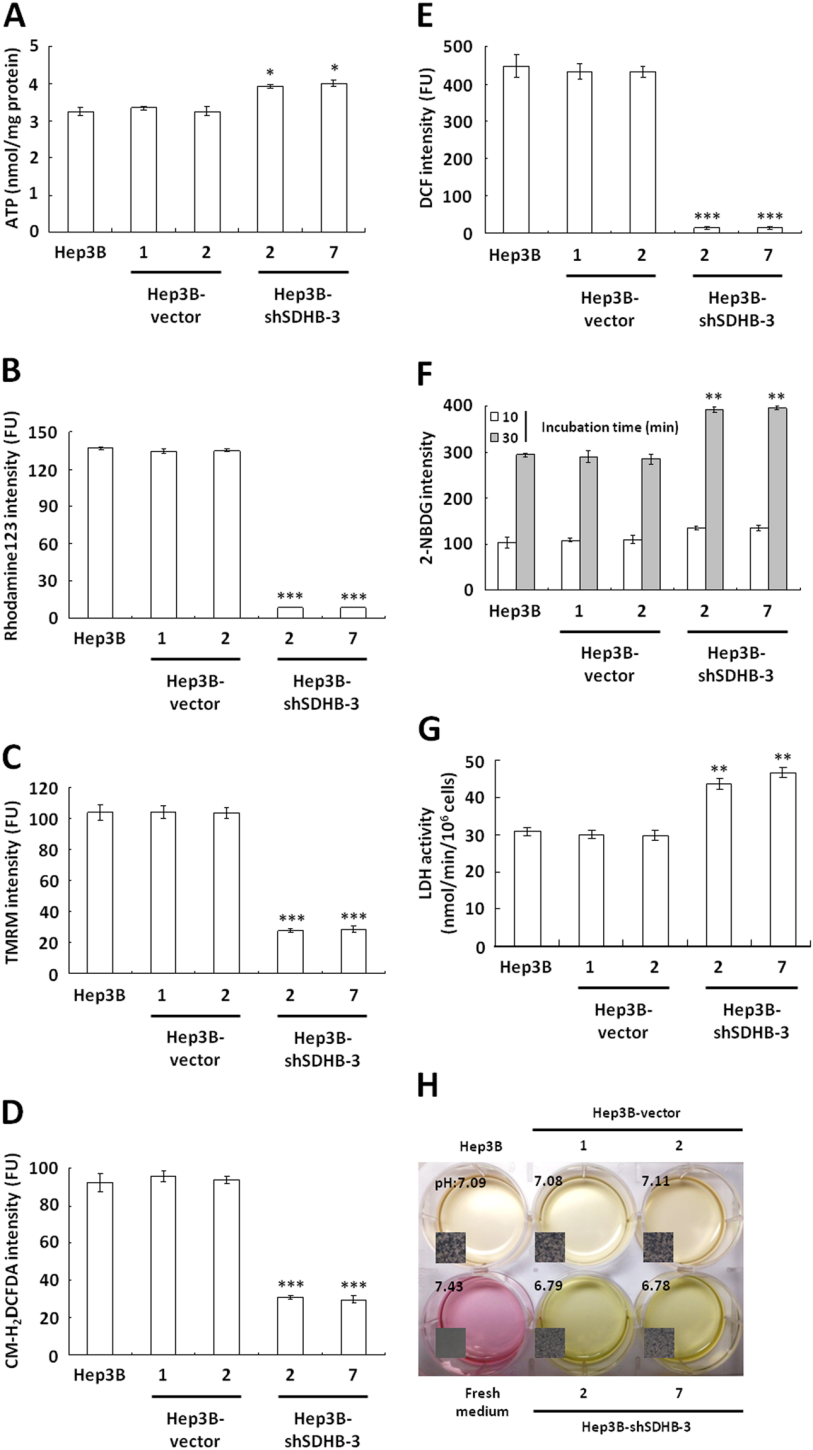
SDHB knockdown causes severe defects in mitochondrial respiration but increases glucose uptake and glycolytic metabolism. (**A**) ATP assay in SDHB-knockdown cells. Total extracts prepared from cells as indicated were subjected to an ATP assay using the ATP Bioluminescence Assay Kit CLSII. (**B**,**C**) Δψ_m_ assay of SDHB-knockdown cells. Cells as indicated were stained with Rhodamine123 and TMRM and then analyzed using a flow cytometer. (**D**,**E**) ROS assay of SDHB-knockdown cells. Cells were treated with CM-H_2_DCFDA and DCF as indicated and subsequently analyzed using a flow cytometer. (**F**) Glucose uptake assay of SDHB-knockdown cells. Cells as indicated were loaded with 2-NBDG and then analyzed using a flow cytometer. (**G**) LDH activity assay of SDHB-knockdown cells. Total extracts prepared from cells were subjected to a LDH activity assay using the CytoTox 96^®^ Non-Radioactive Cytotoxicity Assay. (**H**) Color change and pH measurements of the conditioned media cultured with SDHB-knockdown cells. Cells as indicated were cultured until confluent and then incubated in fresh media for 12 h. The color and pH of the conditioned media were imaged and measured. The *^,^ ** and *** represented *P*-value < 0.05, <0.01 and <0.005, respectively.

To evaluate the effect of SDHB knockdown on bioenergetic metabolism, particularly aerobic respiration, ATP levels in SDHB-knockdown cells were measured using a firefly luciferase ATP assay. Notably, slightly increased ATP levels were detected in the SDHB-knockdown cells compared with the mock- and vector-transfected cells (Fig. 3A). To clarify whether SDHB silencing resulted in this slight ATP increase, the mitochondrial membrane potential (Δψ_m_) was measured to assess respiratory activity in the SDHB-knockdown cells using potentiometric probes, rhodamine 123 and the tetramethylrhodamine methyl ester (TMRM) staining assay. Little staining by either dye was detected in the SDHB-knockdown cells, while strong staining was seen in the mock- and vector-transfected cells (Fig. 3B,C). This suggested that SDHB inhibition impaired TCA cycle progression and reduced mitochondrial respiration. Because reactive oxygen species (ROS) are generated during aerobic respiration, decreased respiratory activity is expected to reduce ROS production. To measure the silencing effect of SDHB on mitochondrial respiration, ROS levels were measured using fluorescent probes, 5-(and -6)-chloromethyl-2′, -7′-diochlorodihydro fluorescein diacetate, acetyl ester (CM-H_2_DCFDA) and dichlorofluorescein (DCF). Low staining intensity with both CM-H_2_DCFDA and DCF was detected in the SDHB-knockdown cells compared with the mock- and vector-transfected cells (Fig. 3D,E). These results clearly demonstrated that loss of SDHB expression impaired mitochondrial function and decreased respiratory activity. Altered SDHB expression impaired mitochondrial respiration and, in turn, activated or upregulated cytosolic glycolysis for cellular ATP production. To examine glucose uptake, the fluorescent glucose analogue 2-[N-(7-nitrobenz-2-oxa-1, 3-diazol-4-yl) amino]-2-deoxyglucose (2-NBDG) was added to the culture media. The SDHB-knockdown cells showed increased glucose uptake compared with the mock- and vector-transfected cells (Fig. 3F). With the increase in glycolysis, acidification of the conditioned media increased due to enhanced lactate dehydrogenase (LDH) activity (Fig. 3G). In addition, compared with the mock- and vector-transfected cells, a color change and decrease in the pH of the conditioned media from the SDHB-knockdown cells were increased greatly (Fig. 3H). Together, these results indicated that silencing of SDHB expression induced the bioenergetic switch from aerobic respiration to glycolytic metabolism.

### Inhibited SDHB expression promotes glycolytic metabolism and induces epithelial-mesenchymal transition (EMT)

**Figure 4:**
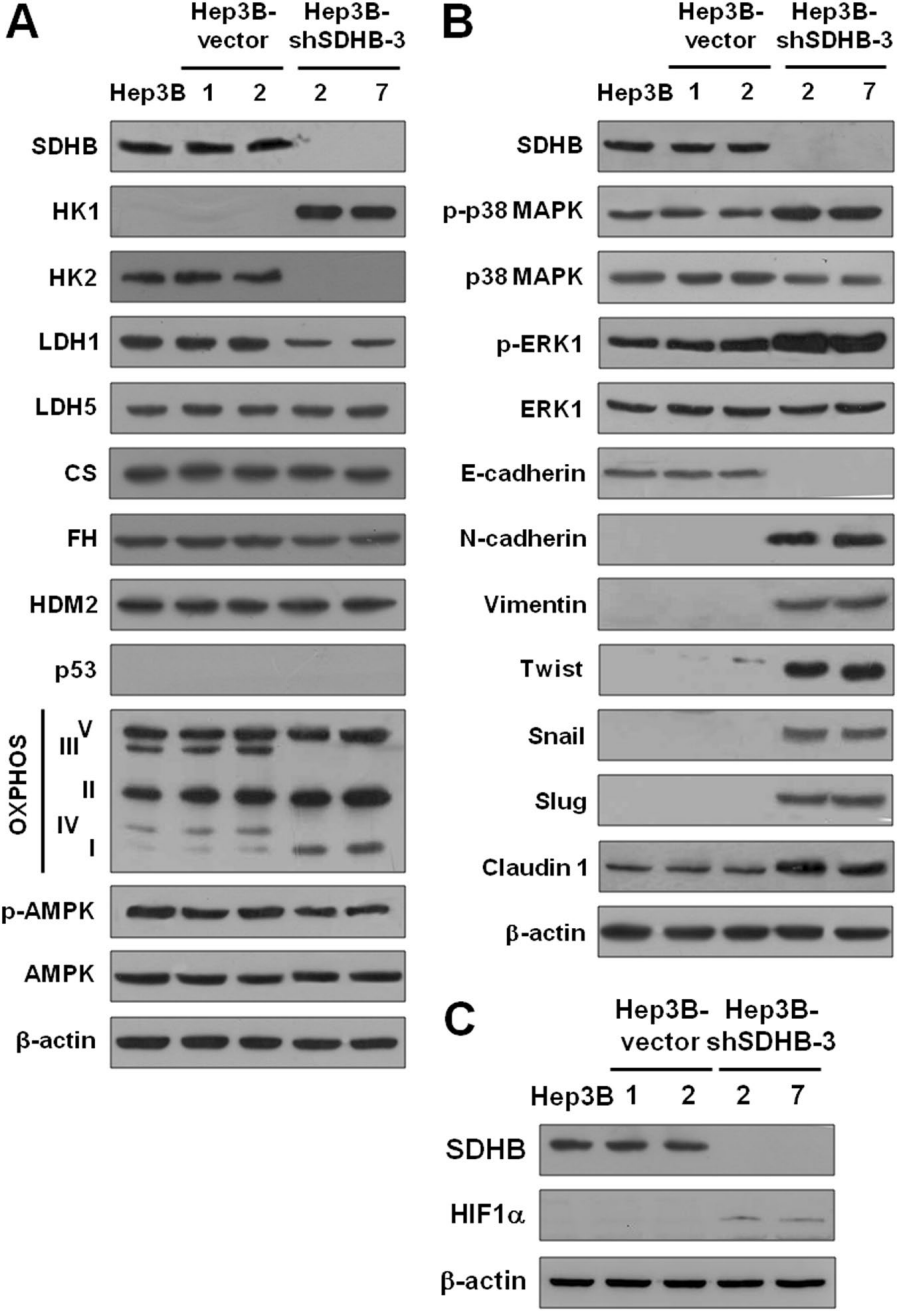
Silencing of SDHB induces the EMT pathway and enhances expression of glycolytic enzymes. (**A**) Western blotting of proteins involved in glycolysis and oxidative phosphorylation in SDHB-knockdown cells. Total proteins isolated from cells were hybridized with antibodies for glycolytic and aerobic respiration enzymes. (**B**) Western blot analysis of EMT-related proteins in SDHB-knockdown cells. Total proteins prepared from cells as indicated were blotted with antibodies against EMT-related proteins, including the indicated cell markers and transcriptional regulators. (**C**) Western blot analysis of HIF1α in SDHB-knockdown cells. Total proteins purified from cells were blotted with antibodies against SDHB, HIF1α and β-actin. The level of β-actin serves as a control for protein loading.

To examine the metabolic changes induced by SDHB knockdown directly, protein expression of the genes involved in bioenergetic metabolism, including the TCA cycle, glycolysis, oxidative phosphorylation (OXPHOS), and homeostatic regulation, in SDHB-knockdown cells were analyzed (Fig. 4A). Expression levels of hexokinase (HK) 2 and LDH1, as well as components of OXPHOS complex III and IV were decreased in the SDHB-knockdown cells compared with the mock- and vector-transfected cells. However, the level of respiratory chain-related proteins, including citrate synthase (CS) and fumarate hydratase (FH), showed no significant changes. In contrast, the level of HK1 was increased greatly. In particular, the activity of AMP activated protein kinase (AMPK), an important bioenergetic homeostatic regulator, was decreased slightly but the activity of p38 mitogen-activated protein kinase (MAPK) was increased in the SDHB-knockdown cells. The activity of extracellular signal-regulated kinase (ERK) was also increased in SDHB-knockdown cells.

To investigate the effects of SDHB silencing on tumor cell growth and motility, EMT-related markers and regulators in the SDHB-knockdown cells were examined using Western blot analysis (Fig. 4B). Compared with the mock- and vector-transfected cells, the protein level of the epithelial marker E-cadherin decreased, while those of the mesenchymal markers vimentin and N-cadherin increased, in the SDHB-knockdown Hep3B cells. The metastasis-related protein claudin-1 was also increased in the SDHB-knockdown cells. In particular, the expression level of the EMT-associated repressors Twist, Slug and Snail increased in the with SDHB knockdown. HIF1α expression also increased in the SDHB-knockdown cells (Fig. 4C).

### Overexpression of SDHB decreases cancer cell proliferation and migration

**Figure 5:**
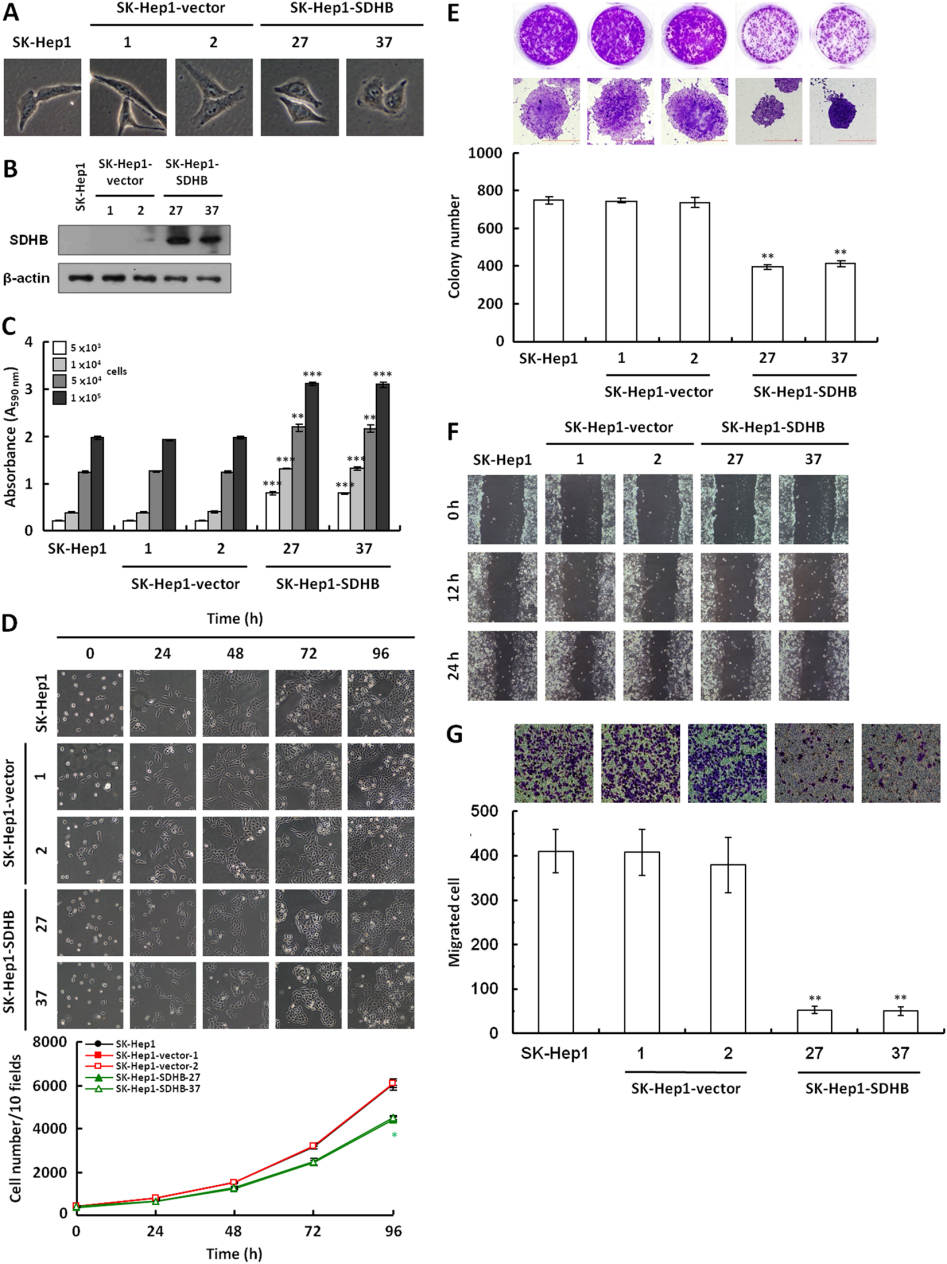
Overexpression of SDHB in HCC SK-Hep1 cells decreases tumor cell proliferation and migration. (**A**) Cell morphology of SDHB-overexpressing cells. Cells were cultured for 48 h and then imaged microscopically. (**B**) Western blot analysis of SDHB protein in SDHB-overexpressing cells. Total proteins prepared from the cells as indicated were blotted with antibodies against SDHB and β-actin. (**C**) SDH activity assay of SDHB-overexpressing cells. An equal number of cells were subjected to a MTT assay according to standard procedures. (**D**) Cell numbers of SDHB-overexpressing cells. Cells as indicated were grown in 6-well plates and 10 random fields were microscopically imaged and counted. (**E**) Colony formation assay of SDHB-overexpressing cells. Cells as indicated were cultured in 6-well plates for 7 days. The colonies were stained and scored. (**F**) Wound healing migration assay of SDHB-overexpressing cells. Cells as indicated were cultured until confluent before being scratched for a wound healing migration assay. (**G**) Boyden chamber migration assay of SDHB-overexpressing cells. Cells as indicated were plated in a Boyden chamber and incubated for 8 h. The migrated cells were stained and counted. The β-actin level serves as control for protein loading. The *^,^ ** and *** represented *P*-value < 0.05, <0.01 and <0.005, respectively.

To examine the role of the SDHB protein in tumor progression or malignancy further, the effect of SDHB overexpression in HCC cells was analyzed using ectopic overexpression vector. For this purpose, several stable SDHB-overexpressing SK-Hep1 cell lines were established (Fig. 5A–C). Cell count results revealed that overexpression of SDHB significantly attenuated cell proliferation compared with the mock and vector-transfected cells (Fig. 5D). In addition, anchorage-dependent clonogenic growth decreased greatly, resulting in reduced colony size and numbers in the SDHB-overexpressing cells (Fig. 5E). In a wound healing migration assay, cell confluence was reached significantly later in the SDHB-overexpressing cells than in the mock and vector-transfected cells (Fig. 5F). A Boyden chamber migration assay revealed that the number of migrated SDHB-overexpressing cells was approximately 10-fold lower than those of the mock and vector-transfected cells (Fig. 5G).

**Figure 6:**
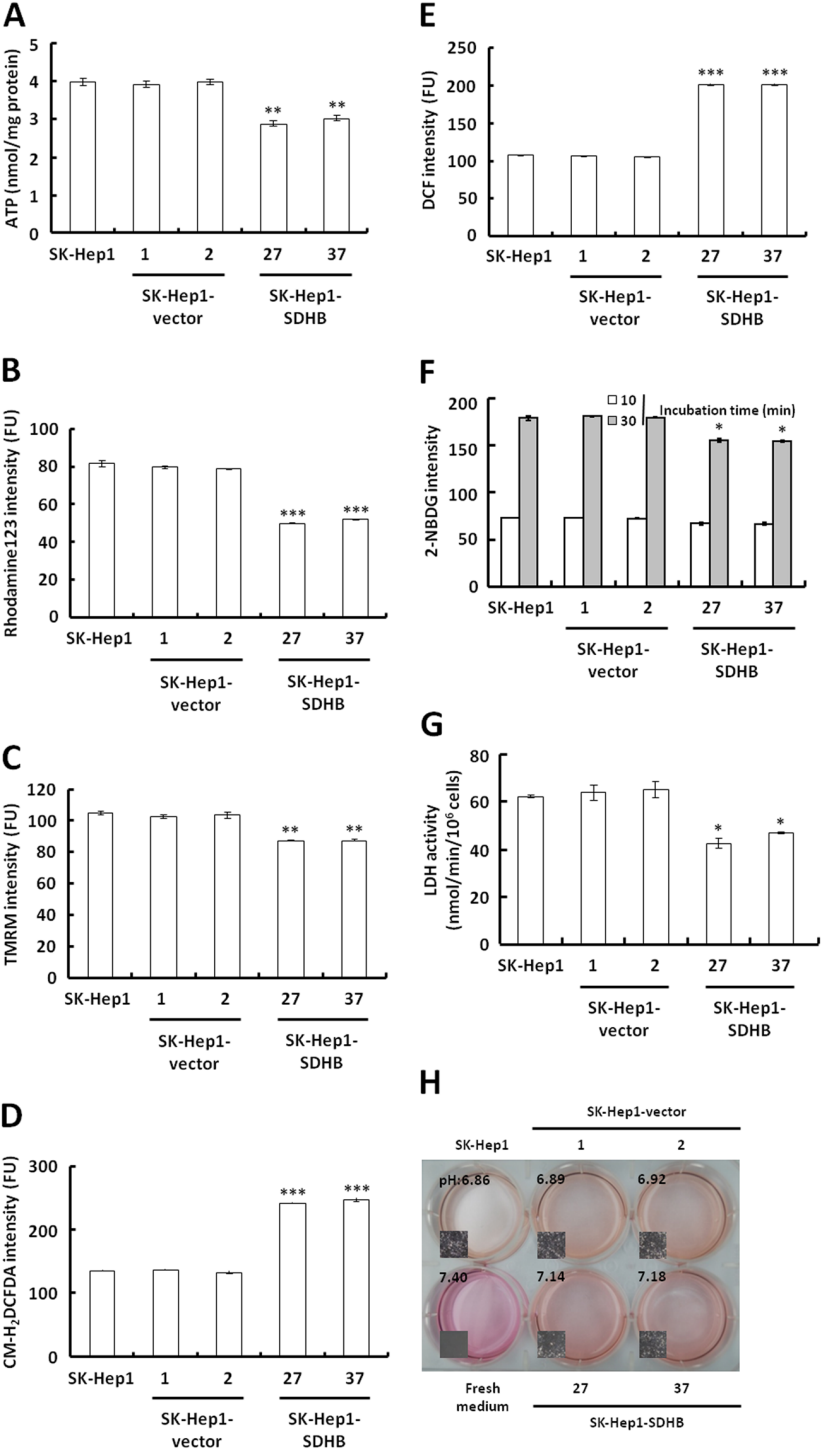
SDHB-overexpressing cells deregulates bioenergetic metabolism. (**A**) ATP assay of SDHB-overexpressing cells. Total extracts were prepared from cells as indicated, and an ATP assay was performed using the ATP Bioluminescence Assay Kit CLSII. (**B** and **C**) Δψ_m_ assay of SDHB-overexpressing cells. Cells were stained with Rhodamine123 and TMRM and analyzed using a flow cytometer. (**D**,**E**) ROS assay of SDHB-overexpressing cells. Cells as indicated were treated with CM-H_2_DCFDA and DCF and subsequently analyzed using a flow cytometer. (**F**) Glucose uptake assay of SDHB-overexpressing cells. Cells as indicated were loaded with 2-NBDG and then analyzed using a flow cytometer. (**G**) LDH activity assay of SDHB-overexpressing cells. Total extracts were prepared from cells and subjected to a LDH activity analysis using a CytoTox 96^®^ Non-Radioactive Cytotoxicity Assay. (**H**) Color change and pH measurement of the conditioned media cultured with SDHB-overexpressing cells. Cells as indicated were cultured until confluent and then incubated in fresh media for 24 h. The color and pH of the conditioned media were imaged and measured. The *^,^ ** and *** represented *P*-value < 0.05, <0.01 and <0.005, respectively.

To evaluate the effect of SDHB overexpression on bioenergetic metabolism, the level of ATP in the SDHB-overexpressing cells was measured by firefly luciferase ATP assay. Notably, markedly reduced ATP levels were observed in the SDHB-overexpressing cells compared with the mock and vector-transfected cells (Fig. 6A). To verify that SDHB overproduction was responsible for the decrease in ATP generation, Δψ_m_ was measured in SDHB-overexpressing cells using potentiometric probes, rhodamine 123, and a TMRM staining assay. Consistent with cellular ATP levels, decreased staining intensity was detected in the SDHB-overexpressing cells compared with the mock and vector-transfected cells (Fig. 6B,C). To further examine the effect of SDHB overexpression on aerobic respiration, ROS formation was measured using the fluorescent probes CM-H_2_DCFDA and a DCF staining assay. In contrast, higher staining intensities with both CM-H_2_DCFDA and DCF were detected in the SDHB-overexpressing cells compared with the mock and vector-transfected cells (Fig. 6D,E).

To analyze the effect of SDHB overexpression on bioenergetic metabolism fully, the glucose uptake ability of SDHB-overexpressing cells was analyzed using a loading assay for the fluorescent glucose analogue 2-NBDG. Compared with the mock and vector-transfected cells, the SDHB-overexpressing cells displayed slightly reduced glucose uptake (Fig. 6F). LDH activity was also decreased in the SDHB-overexpressing cells compared with the mock and vector-transfected cells (Fig. 6G). Furthermore, compared with the mock and vector-transfected cells, there was a less pronounced color change and pH decrease in the conditioned media of the SDHB-overexpressing cells (Fig. 6H). Together, these results indicated that SDHB overexpression in HCC cells not only disrupted TCA cycle progression but also impaired mitochondrial respiration.

## Discussion

Approximately 30% of pheochromocytomas and paragangliomas are hereditary, and nearly half of these cases are caused by germline mutations in the SDH subunits^21,22^. Some SDH-defective tumors exhibit distinct clinical features while others do not. For example, SDH-mutated gastrointestinal stomas showed distinct clinical features, such as onset at a young age, multifocality and a gastric location^23^. Germline mutations of SDHB were also associated with renal cell carcinoma, but not always with different morphologies^24^. SDHB expression has also been investigated in other general solid tumors, such as colon, gastric and breast cancers^25–27^. Levels of SDHB are significantly decreased in human colon cancer tissues, and this is associated with tumor cell dedifferentiation^25^. SDHB showed reduced or loss of expression in 24.5% of gastric cancers but was not associated with clinical parameters, such as tumor invasion and differentiation^27^. A recent study also revealed decreased SDHB expression (48%) in HCC^28^. However, previous studies have not shown the clinical significance of reduced SDHB expression in human HCC. Our study demonstrated that decreased SDHB expression (40.6%) in human HCC was correlated with tumor differentiation, stage (borderline) and overall survival. Furthermore, the SDHB expression pattern in HCC was similar to that of colon cancer.

In this study, several stable SDHB knockdown models were established in Hep3B, HepG2 and Huh7 cells *in vitro*. SDHB-knockdown cells exhibited faster cell proliferation and migration. A SDHB overexpression model was also established in SK-Hep1 cells. SDHB-overexpressing cells showed decreased cell proliferation and migration. Moreover, a SDHB-knockdown cells-transplanted xenograft mouse model also displayed a larger tumor volume than that in mice transplanted with mock or vector-transfected cells. These results differed from the results of a previous study, in which SDHB-knockdown Hep3B cells exhibited slow proliferation and greater tissue adhesion^20^. Although our results were not consistent with these findings, they are compatible with the expression observed in human HCC samples.

Cancers increase the dependency on the glycolytic pathway, in the presence of abundant oxygen, which is needed for energy production and macromolecular synthesis to maintain rapid cell proliferation, compared with the surrounding normal tissues, as first described by Warburg^8,12^. Defects in mitochondrial bioenergetics are a possible mechanism of the Warburg effect. In this study, knockdown of SDHB expression in human HCC cells was associated with a shift in the cellular biogenetics from aerobic respiration to glycolytic metabolism and a simultaneous increase in tumor malignancy. SDHB-knockdown cells exhibited an almost complete loss of the mitochondrial membrane potential and lower levels of ROS, indicating that mitochondrial respiration was severely disrupted. Although increased ROS production has been suggested to contribute to tumorigenesis in SDHB-defective cancers^16^, other studies showed no effect on ROS production after manipulations of several SDH subunits^20,29,30^. Selak *et al*. showed that increased ROS was not required for HIF1α stabilization^31^. A decrease in oxidative phosphorylation also contributed to increased glycolytic activity^32^. A consequence of this metabolic change was increased lactic acid production in tumor cells and consumption of the most abundant extracellular nutrients. Our study showed increases in the acidity of the medium of and glucose uptake in SDHB-knockdown cells. This provides evidence of the Warburg effect, and changes in biogenetic enzymes were directly linked to tumor malignancy *in vitro* and *in vivo*.

Tumor metastasis is the major cause of cancer-associated death. Deregulation of cell migration during tumor progression determines the potential of cancer cells to escape from the primary tumors. A critical consequence of the Warburg effect is increased lactate production in cancer cells, and export of the lactate by transporters, which results in acidification of the microenvironment^33^. A low pH created by extracellular acidification provides a favorable microenvironment for the activation of proteases, including MMPs^34^, urokinase-type plasminogen activator^35^, and cathepsins B^36^, D^37^, and L^38^, which induce extracellular matrix degradation and facilitate tumor cells to metastasize^39^. This study showed that altered cancer cell metabolism also enhanced medium acidification and tumor migration and thus provided the evidence of the Warburg effect directly linked to cancer cell migration both *in vitro* and *in vivo*.

The influence of glycolytic enzymes was also investigated in SDHB-knockdown cells. There was an impact on HK, a key rate-limiting enzyme, after suppression of SDHB expression. HK2 was strongly suppressed, while HK1 expression was increased. HK1 overexpression was also present in a citrate synthase knockdown cell model^40^. HK1 and HK2 share a close physical resemblance, but HK1 exhibits a higher relative affinity for glucose^41^. Another difference of HK1 compared with HK2 is the reduced sensitivity of the former to feedback inhibition by glucose-6-phosphate in the presence of inorganic phosphate^42^. In the SDHB knockdown cells in this study, HK2 expression was suppressed while HK1 expression was enhanced to increase glucose usage, and this correlated with the ability of Akt to maintain the integrity of the outer mitochondrial membrane and to inhibit apoptosis and the release of cytochrome c^43–45^.

In contrast to the SDHB-knockdown cells, cells with SDHB overexpression indeed exhibited a slight change in cell morphology and a clear decrease of both cell growth and migration, suggesting that the SDHB could act as a tumor suppressor. However, the SDHB overexpression caused an unpredictable reduction of both ATP generation and Δψ_m_, but an increase of ROS production. In addition, the SDHB-overexpressing cells also displayed a small decrease of glucose uptake, LDH activity and medium acidification, indicating that overexpression of the SDHB could induce a certain level of reduced glycolytic activity. However, analyzed closely the decreased level of Δψ_m_ in both SDHB-knockdown and -overexpressing cells revealed that the Δψ_m_ reduction in SDHB-overexpressing cells is far small than the decreased level in SDHB-silencing cells. These results suggested that the SDHB overexpression might slightly impair the TCA cycle or OXPHOS and subsequently cause deregulation of glycolytic pathway. In addition, the metabolic alteration in SDHB-overexpressing cells is not phenocopic the energetic aberrance in SDHB-knockdown cells. The possible effect of SDHB overexpression in the TCA cycle or respiratory chain is impaired SDH function or activity by either interfered or improper assembly of SDH holoenzyme.

The loss of or a decrease in SDHB, which plays an important role in the TCA cycle and respiratory chain, induces the bioenergetic switch from mitochondrial respiration to cytosolic glycolysis. These metabolic changes are also associated with tumor dedifferentiation, proliferation, migration and overall patient survival. The dependence of cancer cells on aerobic glycolysis for ATP generation has been exploited as a target for anticancer therapies. Inhibition of glycolysis prevents ATP production and decreases cancer cell growth and proliferation. Thus, attenuation of cancer cell glucose metabolism is a promising and emerging area for the development of novel anticancer therapies^46^. Patients screened for low SDHB expression in tumor tissues and who have a poor prognosis may be treated with anticancer drugs that specifically target the Warburg effect.

## Methods

### Patients and tumor specimens

HCC specimens for immunohistochemical staining, including tumor and matched adjacent non-tumor tissues, were obtained from 96 consecutive patients who underwent surgical resection at Kaohsiung Chang Gang Memorial Hospital from 1987 to 1998. Tumor differentiation was assessed according to the Edmondson-Steiner classification. Grades III-IV were classified as poor differentiation. The pathological stages were classified according to the International Union against Cancer, with minor modifications, as described in a previous study^47^. Overall survival was calculated from the time of surgery to the last follow-up, or death. A total of 15 pairs of fresh frozen HCC and corresponding adjacent non-tumor tissues were also collected. Informed consent was obtained from all patients prior to inclusion in the study. The specimens and experimental protocols were handled and performed in accordance with the approved relevant guidelines and regulations. All samples were subjected to ethical approval by the Human Research Ethics Committee of the Kaohsiung Chang Gang Memorial Hospital. The IRB approval number is 100–4209B.

### Immunohistochemical staining

Paraffin embedded HCC specimens were deparaffinized and blocked with 3% hydrogen peroxide for 10 min. The specimens were then subjected to the antigen retrieval microwave method in 0.01 M citrate buffer for 15 min. The slides were washed with phosphate-buffered saline twice and incubated with anti-SDHB (1:250 dilution; SC-152, Santa Cruz, CA, USA) and anti-CD34 (1:100 dilution; Dakopatts, Copenhagen, Denmark) antibodies for 30 min, followed by horseradish peroxidase conjugated anti-mouse IgG secondary antibody for 30 min, and were then detected using a polymer detection system (catalog no. 87-89431; Zymed, San Francisco, CA, USA). The staining was visualized with 3, 3′-diaminobenzidine tetrahydrochloride (Sigma-Aldrich Chemicals, Saint Louis, MO, USA) in 0.1 M Tris, pH 7.2 containing 0.01% hydrogen peroxide. The section slides were counterstained with Gill hematoxylin, dehydrated, and mounted.

### Western blot analysis

Total protein from the cultured cells was prepared using the following procedure. The cells were harvested by trypsinization, washed with ice-cold PBS, centrifuged at 1,500 × g for 5 min and incubated for 20 min in 50 μL lysis buffer. (62.5 mM Tris-HCl, pH 6.8, 2% sodium dodecyl sulfate (SDS), 5% 2-mercaptoethanol, 10% glycerol). Equal amounts of protein (30 µg) were separated by 10% SDS-polyacrylamide gel electrophoresis and transferred onto a polyvinylidene difluoride membrane. The membrane was blocked with 5% non-fat dried milk in TBST (20 mM Tris-HCl, 150 mM NaCl, and 0.1% Tween 20, pH 7.5) for 2 h and incubated overnight with antibodies against the target proteins at 4 °C. After washing with TBST buffer, membranes were incubated with horseradish peroxidase-conjugated anti-mouse IgG secondary antibody for 1 h at room temperature and detected by an enhanced chemiluminescence detection system. The antibodies used were goat polyclonal HK2 (C-14, Santa Cruz Biotechnology, Santa Cruz, CA, USA), mouse monoclonal anti-SDHB (FL-280, Santa Cruz Biotechnology), mouse anti-FH (J-13, Santa Cruz Biotechnology), mouse anti-p53 (DO-1, Santa Cruz Biotechnology), mouse anti-HK1 (G-1, Santa Cruz Biotechnology), mouse anti-HDM2 (Human MDM2, SMP14, Santa Cruz Biotechnology), mouse monoclonal anti-β-actin (Santa Cruz Biotechnology), mouse anti-CS (D3G4, Chemicon International, Temecula, CA, USA), mouse anti-LDH1 (H10, Santa Cruz Biotechnology) and sheep anti-LDH5 (LDHV, Abcam, Cambridge, USA).

### Cell culture

Human HCC cell lines HepG2 and Hep3B were obtained from the Bioresource Collection and Research Center (Hsinchu, Taiwan), and SK-Hep 1 was purchased from the American Type Culture Collection (ATCC; Rockville, MD, USA). Huh7 cells were obtained from the Japanese Collection of Research Bioresource (JCRB) Cell Bank (Tokyo, Japan). J2 and J5 cells were provided by Dr. J.-H. Chung (Chang-Gang Memorial Hospital, Kaohsiung, Taiwan). The Mahlavu cell line was supplied by Dr. M.-H. Tai (National Sun Yat-sun University, Kaohsiung, Taiwan). Cells were cultured in growth medium containing 10% fetal bovine serum, penicillin (100 IU/mL) and streptomycin (100 μg/mL). They were incubated at ambient oxygen concentration in the presence of 5% CO_2_ at 37 °C.

### RNAi expression vector

Plasmid vectors were constructed using standard molecular cloning techniques or polymerase chain reaction (PCR)-based strategies. Oligonucleotides used in this study were purchased from local commercial suppliers. The stable shRNA expression vector pSUPER/Hyg^r^ was constructed by inserting the hygromycin resistance gene expression cassette isolated from pDsRed2-N1 into pSUPER^48^. To design an effective shRNA expression vector, the shRNAs were screened and constructed using a fully robust and comparative siRNA validation system^49^. In general, the shRNA expression vectors were constructed by ligation of an annealed oligonucleotide duplex into *Bgl*II/*Hind*III restriction enzyme sites within the pSUPER/Hyg^r^ vector. The following oligonucleotides were used for cloning of pshSDHB: shSDHB-F, 5′-gatccccCCTATCGCTGGATGATTGAttcaagagaTCAATCATCCAGCGATAGGttttttggaaa-3′ and shSDHB-R, 5′-agcttttccaaaaaCCTATCGCTGGATGATTGAtctcttgaaTCAATCATCCAGCGATAGGggg-3′.

### SDHB overexpression construct

Plasmid vectors were constructed using standard molecular cloning techniques or PCR-based strategies. The oligonucleotides used in this study were purchased from local commercial suppliers. The stable expression vector pCMV-SDHB-AC/Neo^r^ was constructed by inserting the neomycin resistance gene expression cassette. The following oligonucleotides were used for cloning pCMV-SDHB-AC. After transfection, conjugation vectors were selected by neomycin resistance.

### MTT cell growth assay

Cells were loaded into 96-well plates at the number indicated in 200 µL growth medium. The cells were treated with 10 µL MTT solution (5 mg/mL) for 6 h, disrupted in 200 µL dimethyl sulfoxide (DMSO), and measured at 590 nm using an ELISA reader (VERSA_max_ tunable microplate reader, Molecular Dynamics, Sunnyvale, CA, USA).

### Cell number counting

HCC cells were seeded into a 6-well plate (1 × 10^5^/well). Viable cell numbers and spreading numbers were calculated as averages of 10 counts of total numbers every 24 h. The medium was changed every 48 h.

### Colony formation assay

Cells were plated into 6-well plates at a density of 2 × 10^3^/well. After 6 days, the colonies were stained with crystal violet for 24 h, rinsed with deionized distilled water, photographed using a Nikon D80 digital camera (10 Mega-pixel; Nikon Corp., Tokyo, Japan), and scored using AlphaEase FC software (Alpha Innotech Inc., San Leandro, CA, USA).

### Wound healing assay

HCC cell lines (1 × 10^5^/2 mL) were grown in 6-well plates until fully confluent with medium changes at 48 h intervals and then scratched using a plastic 1 ml micropipette tip. The wound width was photographed using an inverted phase-contrast microscope every 12 h until complete healing.

### Boyden chamber assay

Cells were cultured in 10 cm Petri dishes to 70–80% confluence and harvested using 0.1% trypsin (Cambrex, East Rutherford, NJ, USA). A total of 2.5 × 10^4^ cells were seeded onto 8-μm pore-size polycarbonated filters (Nucleopore Crop., Pleasanton, CA, USA) in a 48-well Boyden chamber. The chemotactic migration of cells was induced by 10% fetal calf serum (FCS) in the lower chamber. After 6 h of incubation, the cells on the upper surface were removed using a cotton bud. The remaining invading cells were fixed and stained with Liu’s stain for 1 h at room temperature. Finally, the invaded cells were photographed and analyzed using an inverted phase-contrast microscope. Experiments were repeated three times.

### Xenograft models

NOD/SCID mice were maintained according to the Institutional Animal Care and Use Committee of the National Chung Kung University, Tainan, Taiwan. Male mice 8 to 12 weeks old were used in this study. Tumors were produced by subcutaneously injecting 5 × 10^6^ Hep3B–shSDHB cells. Tumor dimensions were measured every 3–5 days using vernier calipers. The tumor volume was calculated using the following formula: tumor volume (mm^3^) = length × width^2^ × 0.52. After 20 or 60 days, mice were euthanized. The tumors were removed and photographed. Tumor metastases were generated by intravenously injecting 5 × 10^5^ Hep3B-shSDHB cells into the tail vein of NOD/SCID mice. After 20 days, mice injected with Hep3B-shSDHB cells appeared to be sick, so all mice were euthanized and examined for tumor metastases. Their organs, particularly lungs and hearts, were removed and fixed in formalin, and processed for histological and immunohistochemical analyses. The methods used in animal experiments were performed in accordance with the relevant guidelines. All animal studies were approved by the Institutional Animal Care and Use Committee (IACUC) of the National Cheng Kung University, Tainan, Taiwan. The IACUC approval number is 103113.

### Firefly luciferase ATP assay

Total cellular ATP was measured using the ATP Bioluminescence Assay Kit CLSII (Roche Applied Science, Mannheim, Germany) according to the manufacturer’s instructions. The luminescent signals were quantified using a bioluminescence detection system (Lumat LB 9507 luminometer, Berthold Technologies, Bad Wildbach, Germany).

### Mitochondrial membrane potential (Δψm) assay

The Δψ_m_ level corresponds to the cellular respiration activity. Cells were seeded into 6-well plates at a density of 1 × 10^5^/well. After 48 h, the cells were treated with potentiometric probes, 200 nM rhodamine 123 (Molecular Probes Inc., Eugene, OR, USA) and 150 nM tetramethylrhodamine methyl ester (TMRM; Molecular Probes Inc.) for 30 min in culture medium. The stained cells were analyzed quantitatively by flow cytometry using a FACSCalibur^TM^ flow cytometer (Beckton Dickinson, San Jose, CA, USA).

### ROS measurement

Cells were inoculated into 6-well plates at a density of 1 × 10^5^/well. After 48 h, the cells were loaded with 1 µM 5–6 chloromethyl-2′,7′-dichlorodihydro-fluoresceine diacetate acetyl ester (CM-H_2_DCFDA; Molecular Probes Inc.) and 10 µM 2′,7′ dichlorodihydrofluorescein diacetate (DCF; Sigma, St. Louis, USA) for 30 min in phenol red–free culture medium. The stained cells were analyzed quantitatively by flow cytometry using a FACSCalibur^TM^ flow cytometer (Beckton Dickinson).

### Glucose uptake assay

Cells were seeded into 6-well plates at a density of 1 × 10^5^/well. After 48 h, the cells were pre-incubated in glucose-free Krebs-Ringer bicarbonate (KRB) buffer (129 mM NaCl, 5 mM NaHCO_3_, 4.8 mM KCl, 1.2 mM KH_2_PO_4_, 1.0 mM CaCl_2_, 1.2 mM MgSO_4_, 10 mM HEPES, and 0.1% BSA; pH 7.4) for 15 min and then incubated in fresh KRB buffer supplemented with 600 μM 2-N-(7-nitrobenz-2-oxa-1,3-diazol-4-yl) amino-2-deoxy-D-glucose (2-NDBG; Molecular Probes Inc.), a D-glucose fluorescent analogue, and 3.3 mM glucose for 10 and 30 min. The stained cells were analyzed quantitatively by flow cytometry using a FACSCalibur^TM^ flow cytometer (Beckton Dickinson).

### LDH activity assay

Cells were inoculated into 96-well plates at a density of 1 × 10^4^/well in 200 µL culture medium for 2 h. The cells were lysed and LDH activity quantified by the CytoTox 96^®^ Non-Radioactive Cytotoxicity Assay (Promega, Fitchburg, WI, USA) according to the manufacturer’s instructions.

### pH measurement

Cells were seeded into 6-well plates at a density of 1 × 10^5^/well. The cells were cultured until confluent and then incubated in fresh culture medium for 12 or 24 h. The colors and pH values of the conditioned media were photographed using a Nikon D80 digital camera (Nikon Corp.) and measured using a FE20-FiveEasy^TM^ pH meter (Mettler Toledo, Zchwerzenbach, Switzerland).

### Statistical analyses

Results were presented as mean ± SD unless otherwise stated. Continuous variables were compared using independent Student’s test. Categorical variables were compared using chi-square or Fisher’s exact test. Kaplan-Meier estimation and log rank test were used to examine the disease free survival and overall survival. Cox regression analysis was used for univariate and multi-variate analyses. All statistical analyses were performed using the GraphPad Prism 5 Software. The difference between two groups was recognized statistically significant when *P*-value was < 0.05. The *, ** and *** represented *P*-value < 0.05, <0.01 and <0.005, respectively.

## Electronic supplementary material

Decreased succinate dehydrogenase B in human hepatocellular carcinoma accelerates tumor malignancy by inducing the Warburg effect
