# Supplementary material for: Decreased succinate dehydrogenase B in human hepatocellular carcinoma accelerates tumor malignancy by inducing the Warburg effect
Source: Sci Rep. 2018 Feb 15;8:3081. doi: 10.1038/s41598-018-21361-6 (PMC5814459; doi:10.1038/s41598-018-21361-6)

**Supplementary Information**

**Decreased succinate dehydrogenase B in human hepatocellular carcinoma accelerates tumor malignancy by inducing the Warburg effect**

**Po-Lin Tseng^1,3,#^, Wei-Hsuan Wu^2,#^, Tsung-Hui Hu^3^, Chih-Wei Chen^4,8,9^, Hung-Chi Cheng^2^, Chien-Feng Li^5^, Wen-Hui Tsai^6^, Hui-Ju Tsai^2^, Meng-Che Hsieh^2^, Jiin-Haur Chuang^7^ and Wen-Tsan Chang^2,4,*^**

^1^Graduate Institute of Clinical Medical Sciences, College of Medicine, Chang Gung University, Taoyuan 302, Taiwan; ^2^Department of Biochemistry and Molecular Biology, College of Medicine, National Cheng Kung University, Tainan 701, Taiwan; ^3^Division of Hepato-Gastroenterology, Department of Internal Medicine, Chang Gung Memorial Hospital–Kaohsiung Medical Center, Kaohsiung 833, Taiwan; ^4^Institute of Clinical Medicine, College of Medicine, National Cheng Kung University, Tainan 701, Taiwan; ^5^Department of Pathology, Chi Mei Foundation Medical Center, Tainan 710, Taiwan; ^6^Department of Pediatrics, Chi Mei Foundation Medical Center, Tainan 710, Taiwan; ^7^Department of Pediatric Surgery, Kaohsiung Chang Gung Memorial Hospital–Kaohsiung Medical Center, Kaohsiung 833, Taiwan; ^8^Department of Surgery, Chi Mei Foundation Medical Center, Tainan 710, Taiwan; ^9^Department of Occupational Safety and Health/Institute of Industrial Safety and Disaster Prevention, College of Sustainable Environment, Chia Nan University of Pharmacy and Science, Tainan 717, Taiwan

^#^These authors contributed equally to this work.

*Correspondence and requests for materials should be addressed to W.-T.C. (wtchang@mail.ncku.edu.tw).

**Supporting Figure Legends**

**Figure S1**｜**Knockdown of SDHB in HCC HepG2 cells largely increases tumor cell growth and migration.** (A) Cell morphology of SDHB-knockdown cells. Cells were cultured for 48 h and imaged microscopically. (B) Western blot analysis of SDHB protein in SDHB-knockdown cells. Total proteins isolated from cells as indicated were probed with antibodies against SDHB and β-actin. (C) SDH activity assay in SDHB-knockdown cells. 5 × 10^3^ cells as indicated were carried out a MTT assay according to standard protocols. (D) SDHB-knockdown cell counts. Cells as indicated were cultured in 6-well plates, and 10 random fields were imaged microscopically. Cells in the 10 fields were scored. (E) Colony formation assay of SDHB-knockdown cells. Cells as indicated were seeded into 6-well plates for 10 days. The colonies appeared were stained and scored. (F) Wound healing migration assay of SDHB-knockdown cells. Cells as indicated were grown until confluent before a scratch wound healing migration assay was performed. (G) Boyden chamber migration assay of SDHB-knockdown cells. Cells as indicated were seeded in a Boyden chamber and incubated for 48 h. The migrated cells were stained and counted. The β-actin level serves as a control for protein loading. The * and ** represented *P*-value ＜ 0.05 and ＜ 0.01, respectively.

**Figure S2**｜**Knockdown of SDHB in HCC Huh7 cells strongly enhances tumor cell proliferation and movement.** (A) Cell morphology of SDHB-knockdown cells. Cells were grown for 48 h and imaged microscopically. (B) Western blot analysis of SDHB protein in SDHB-knockdown cells. Total proteins prepared from cells as indicated were blotted with antibodies against SDHB and β-actin. (C) SDH activity assay in SDHB-knockdown cells. 5 × 10^3^ cells as indicated were subjected to a MTT assay according to standard procedures. (D) SDHB-knockdown cell counts. Cells as indicated were cultured in 6-well plates, and 10 random fields were imaged microscopically. Cells in the 10 fields were scored. (E) Colony formation assay of SDHB-knockdown cells. Cells as indicated were plated into 6-well plates for 10 days. The colonies obtained were stained and scored. (F) Wound healing migration assay of SDHB-knockdown cells. Cells as indicated were grown until confluent before a scratch wound healing migration assay was carried out. (G) Boyden chamber migration assay of SDHB-knockdown cells. Cells as indicated were plated in a Boyden chamber and incubated for 12 h. The migrated cells were stained and counted. The β-actin level serves as a control for protein loading. The ** and *** represented *P*-value ＜ 0.01 and ＜ 0.005, respectively.


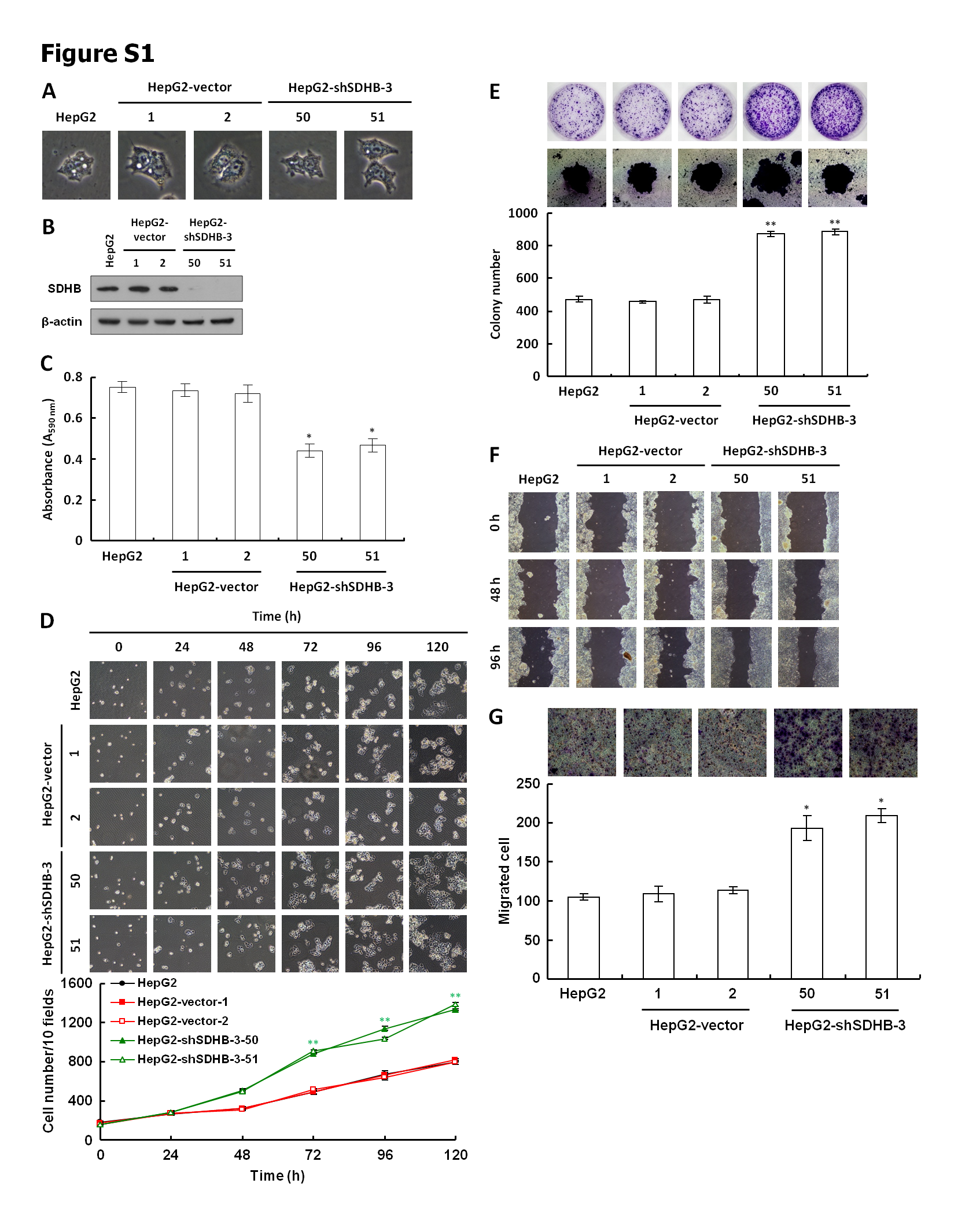


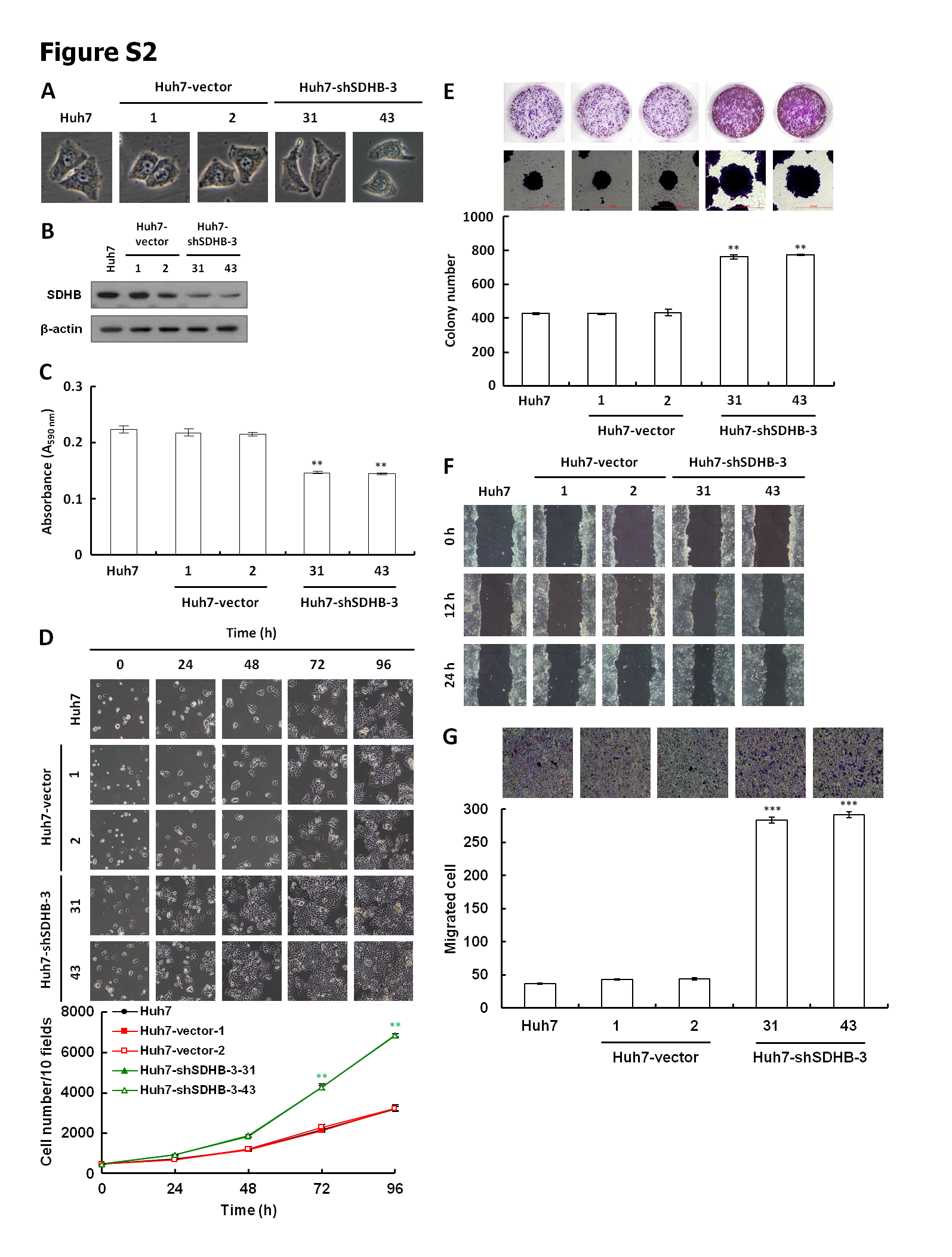

Supplement: Supplementary file 1 — Decreased succinate dehydrogenase B in human hepatocellular carcinoma accelerates tumor malignancy by inducing the Warburg effect [file 41598_2018_21361_MOESM1_ESM.doc]
